# Allelic incompatibility can explain female biased sex ratios in dioecious plants

**DOI:** 10.1186/s12864-017-3634-5

**Published:** 2017-03-23

**Authors:** Pascal Pucholt, Henrik R. Hallingbäck, Sofia Berlin

**Affiliations:** 1 Department of Plant Biology, Uppsala BioCenter, Linnean Centre for Plant Biology, Swedish University of Agricultural Sciences, P.O. Box 7080, SE - 75007 Uppsala, Sweden; 2 Department of Forest Genetics and Plant Physiology, Umeå Plant Science Center, Swedish University of Agricultural Sciences, SE - 90183 Umeå, Sweden

**Keywords:** Sex determination, Sex ratio, Willows, Genotyping-by-sequencing, Dioecy

## Abstract

**Background:**

Biased sex ratios are common among dioecious plant species despite the theoretical prediction of selective advantage of even sex ratios. Albeit the high prevalence of deviations from even sex ratios, the genetic causes to sex biases are rarely known outside of a few model species. Here we present a mechanism underlying the female biased sex ratio in the dioecious willow species *Salix viminalis*.

**Results:**

We compared the segregation pattern of genome-wide single nucleotide polymorphism markers in two contrasting bi-parental pedigree populations, the S3 with even sex ratio and the S5 with a female biased sex ratio. With the segregation analysis and comparison between the two populations, we were able to demonstrate that sex determination and sex ratio distortion are controlled by different genetic mechanisms. We furthermore located the sex ratio distorter locus to a Z/W-gametologous region on chromosome 15, which was in close linkage with the sex determination locus. Interestingly, all males in the population with biased sex ratio have in this sex ratio distorter locus the same genotype, meaning that males with the Z_1_/Z_3_-genotype were missing from the population, thereby creating the 2:1 female biased sex ratio.

**Conclusions:**

We attribute the absence of Z_1_/Z_3_ males to an allelic incompatibility between maternally and paternally inherited alleles in this sex ratio distorter locus. Due to the tight linkage with the sex determination locus only male individuals are purged from the population at an early age, presumably before or during seed development. We showed that such allelic incompatibility could be stably maintained over evolutionary times through a system of overdominant or pseudooverdominant alleles. Thus, it is possible that the same mechanism generates the female biased sex ratio in natural willow populations.

**Electronic supplementary material:**

The online version of this article (doi:10.1186/s12864-017-3634-5) contains supplementary material, which is available to authorized users.

## Background

Sexual reproduction is a fundamental biological process that creates novel and beneficial allelic combinations by bringing together and mixing maternal and paternal gametes [1]. In most animals, sexual reproduction has led to the evolution of separate sexes, whereas among angiosperm plants only about 5% are dioecious with female and male flowers on separate plants [2]. Dioecious species are found in multiple plant lineages, suggesting that separate sexes have repeatedly evolved from co-sexual ancestors [3]. In organisms with separate sexes, natural selection is expected to maintain a balanced sex ratio, provided that the cost of producing male and female offspring is equal [4–6]. Many species however, display more or less stable female or male biased sex ratios [7–9], governed by often unknown genetic or ecological mechanisms. The limited knowledge on causes of biased sex ratios partly owes to the difficulty in determining the phenotypic sex in juvenile and non-reproducing individuals, and therefore there is a lack of knowledge on when in an organism’s life cycle the sex bias is introduced. Molecular and cytological methods can in some cases however be used for determining sex also in non-reproducing individuals [10–13].

Biased progeny sex ratios can for example arise from sex chromosome meiotic drive, which is the non-Mendelian segregation of sex determination alleles [14]. Sex chromosome meiotic drive is common among *Drosophila* species, although it has rarely been documented in plants [14]. X chromosome meiotic drive is however involved in creating female biased sex ratios in *Silene latifolia* [15–17]. Pollen competition (certation) is another phenomenon that can distort progeny sex ratios in plants and has been demonstrated to lead to female biased sex ratios in the male heterogametic species *Rumex nivalis* [12] as well as in *Silene latifolia* [15–17]. Sex-biased mortality can furthermore influence both progeny and adult sex ratios, which can be a consequence of both genetic and ecological differences between the sexes. Examples are genotype specific mortality of zygotes, seeds or seedlings possibly arising from allelic or genotypic incompatibilities at key loci. For instance, a single-locus self incompatibility system has been suggested to have caused male-biased sex-ratios in the adrodioecious shrub species *Phillyrea angustifolia* [18]. Allelic/genotypic incompatiblitites can also possibly act prezygotically, preventing fusion of sperm and egg carrying certain alleles at key loci. A similar mechanism is the well-known phenomenon self-incompatibility in plants, which prevents self fertilization in co-sexual species and can, if acting late, even prevent the embryonal development of zygotes generated by self mating or by the mating of close relatives [19]. Furthermore, in species with heteromorphic sex chromosomes, the theoretical background to the ‘unguarded sex chromosome hypothesis’ predicts that mortality will be higher in the heterogametic sex compared to the homogametic sex because the heterogametic sex will be hemizygous for a large number of loci and will be unable to mask the expression of recessive deleterious alleles [8]. On the other hand, it has been demonstrated that the presence of recessive deleterious alleles, in combination with systematic inbreeding, can cause a greater mortality in the homogametic sex [20]. These results suggest that hemizygous individuals may carry a genetic factor that consistently mask deleterious alleles from expression.

Willows in the *Salix* genus are long-lived woody angiosperm shrubs or trees. Most willows are dioecious, with female and male flowers on separate individuals. Interestingly, most lineages in the Salicaceae family are dioecious, indicating that dioecy evolved early in this clade [21]. Furthermore, the majority of willow species are wind- and insect pollinated, can propagate both sexually and clonally and have wind-dispersed seeds. Most willows are diploid with a basis chromosome number of *n* = 19, although rare cases of polyploids have been encountered [22, 23]. Willow species studied thus far have largely undifferentiated, homomorphic sex chromosomes with narrow sex associated regions on chromosome 15 [24–26]. A female specific haplotype and high heterozygosity levels in the sex associated region suggests that females are the heterogametic sex with one Z and one W allele in a sex determination locus, whereas males have two Z alleles [26]. There is thus far no indications that females are hemizygous over any long distances as they were perceived as heterozygous at all genetic markers linked to the region [26].

Interestingly, many willow species display distinct female biased sex ratios in wild populations [27–34] (e.g., *S. viminalis* [35], *S. repens* [31] and five species of alpine willows [32]). Until now, mechanisms creating the sex bias in willows are unknown. It is however possible to dismiss some of the mechanisms that are known to create sex biases in other systems because they would not create female biased sex ratios in organisms with female heterogamety with homomorphic sex chromosomes. Z chromosome meiotic drive would for example lead to male biased sex ratios and certation is unlikely in female heterogametic species with homomorphic sex chromosomes since the male gamete (pollen) is not determining the sex of the offspring. Most likely some mechanism involving sex biased mortality is operating. The main aim of the present study is therefore to identify underlying genetic mechanisms associated with the female biased sex ratios in willows. In addition, we confirm previous findings of single locus sex determination and female heterogamety and finally we present genetic models that would be able to explain the sex bias while simultaneously be evolutionary stable. To achieve this, we studied two pedigree populations of *Salix viminalis*, one that displays an even sex ratio and one that displays a female biased sex ratio by performing segregation analyses using genome-wide single nucleotide polymporphism (SNP) markers.

## Results

In this study, genotype data from the *S. viminalis* S5 pedigree population was analyzed. The S5 population is composed of 182 females and 89 males, and therefore displays an overall 2:1 female biased sex ratio. Additionally, data from the S3 pedigree population were re-analyzed [26, 36–38]. The analyzed S3 population consists of 265 females and 251 males, hence displaying an even sex ratio. Both populations share the same male parent (81084), whereas the female parents differ (78021 and 78195 for S5 and S3 populations respectively). In a previous study, coancestry coefficients, estimated from molecular marker data [39], suggested the parent pairs to be close to unrelated (0.0420 and 0.0598 for the S5 and S3 parent pairs respectively), thus implying low inbreeding in the S5 and S3 offspring.

### A maternally segregating sex determination locus located on chromosome 15

**Fig. 1:**
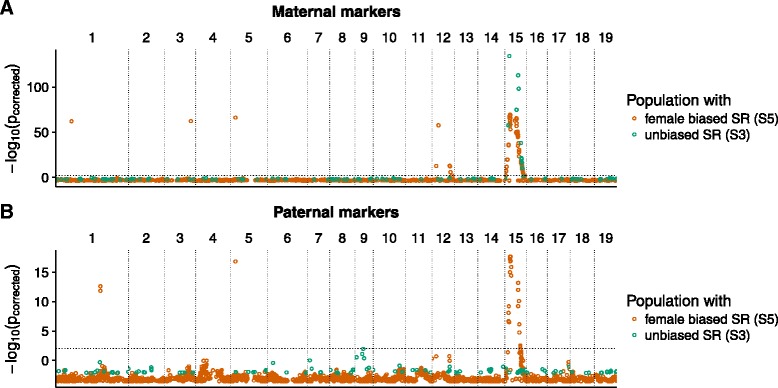
*p*-values for distorted marker alleles between male and female offspring. The markers were assigned genomic positions based on BLAST searches against the *P. trichocarpa* genome. **a** Markers that were heterozygote only in the mother and thus reflect inheritance of maternal alleles. **b** Markers that were heterozygote only in the father and thus reflect inheritance of paternal alleles. Datapoints in turquoise are based on the S3 population with unbiased sex ratio, datapoints in orange are based on the S5 population with female biased sex ratio

In a female heterogametic sex determination system, maternal alleles are expected to co-segregate with the sex of the offspring. We therefore studied the segregation distortion between male and female offspring for all maternal markers to identify putative female heterogametic regions. For both the S5 and the S3 population, the majority of significantly (corrected *p*-value < 0.05) distorted maternal markers were located on chromosome 15 (72% and 100% respectively, see Table 1 and Additional file 1). The most significantly distorted markers from both populations co-located, suggesting a shared location of a female heterogametic region on chromosome 15 that likely harbours a common sex determination (SD) locus (see Fig. 1). Since the populations differ in their sex ratio this suggests that sex determination and sex ratio distortion are two separate processes.

**Table 1:** Number of significantly distorted genetic markers grouped by genomic position. Maternal markers are such markers that are heterozygous in the mother, paternal markers are heterozygous in the father. Population S3 shows unbiased sex ratio while population S5 has a 2:1 female:male sex ratio

	maternal markers		paternal markers	
	Pop S5	Pop S3	Pop S5	Pop S3
Chromosome 15	63	13	28	0
unknown chromosome	14	0	7	0
any other chromosome	10	0	3	2

### Paternal alleles linked to sex ratio distortion

To identify genomic regions associated with sex ratio bias, we searched for distorted paternal markers in both the S3 and the S5 population. Of the 2,765 paternally segregating markers in the S5 population, 38 were significantly distorted between male and female offspring (corrected *p*-value < 0.05, Additional file 1) and for the most significantly distorted markers, one allele was present in 50% of the female offspring but 100% of the male offspring hence expressing an overall 2:1 ratio. The majority of these markers were located on chromosome 15 (73%, see Table 1 and Additional file 1), in close proximity to the maternally segregating SD locus (Fig. 1). In contrast to this observation, paternal markers in the S3 population showed limited segregation distortion. In fact, only two of 137 paternal markers were significantly distorted (see Fig. 1 and Table 1). These markers were located on chromosome 9 and showed only weak deviations from the 50% allele frequency in males. Segregation distortion of paternal markers were thus primarily found in the S5 population with biased sex ratio, suggesting that these markers are associated with a sex ratio distorter (SR) locus on chromosome 15, in close proximity to the SD locus.

### Absence of a male genotype class explains the female biased sex ratio

**Fig. 2:**
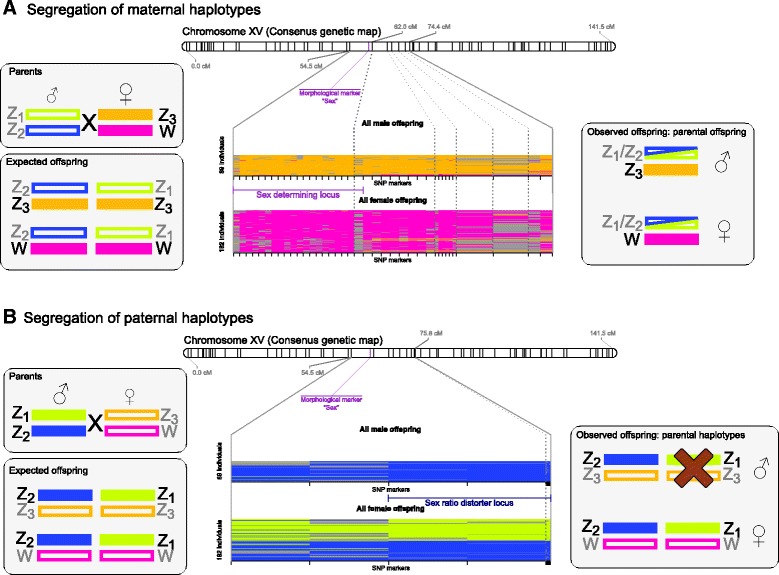
Segregation of marker alleles and inferred haplotypes in proximity to the sex determination and sex ratio distorter (SR) loci on chromosome 15. The inheritance of maternal (**a**) and paternal (**b**) haplotypes are presented (*left*) and the haplotypes that are present in the population (one horizontal line per individual) are shown divided by male and female offspring (*center*). The mode of segregation of the non-recombined parental haplotypes for the observed offspring is depicted schematically (*right*). **a** All female offspring inherit the W-gametolog. **b** The analysis of paternally segregating haplotypes shows that no males possess the Z_1_/Z_3_-genotype in the SR locus. Recombination events are shown as change in color in the line representing an individual. Missing data: gray

To more precisely map the SD and the SR loci and to detect putative recombination points, we inferred maternal, paternal and recombinant offspring haplotypes based on the genotypes at markers surrounding the SD and SR loci on chromosome 15 in the S5 population. To obtain the order of markers, we generated a dense genetic linkage map covering the whole *S. viminalis* genome in 19 linkage groups (Additional file 2). The finding that the morphological marker for sex mapped at 60.7 cM on the linkage map of chromosome 15 supports the finding from the segregation analysis of a sex determination locus on this chromosome. The linkage group that represents chromosome 15 contained 69 markers over the distance of 141.5 cM. We focused our analyses to the region of strong segregation distortion of both paternal and maternal markers from 54.5 to 75.8 cM. To increase the marker density in this region, markers that showed a distorted segregation but were not previously integrated into the map, were anchored through the *S. purpurea* genome assembly. All four parental haplotypes were obtained by inferring the phase from the genotypes at each marker locus. As expected, all female offspring carried one of the maternal haplotypes and all male offspring carried the other maternal haplotype (W and Z_3_ in Fig. 2). As a low number of recombinants were observed towards one end of the region, the SD locus is likely located in the non-recombining region, assuming the absence of recombination between the Z- and W-gametolog in females (which coincides with the “sex” marker). The two paternal haplotypes (Z_1_ and Z_2_) were found in approximately equal frequency among the female offspring (95 vs. 87) including several paternal recombinants that showed no significant bias to a certain haplotype. Interestingly, no male with the whole paternal Z_1_-haplotype was found and only a few recombinants with parts of the Z_1_-haplotype towards one end of the analyzed region were encountered (Fig. 2b). For the S3 population with even sex ratio, no such effect was observed, although the populations share the same male parent (see Fig. 1), indicating that this effect is not solely dependent on the paternal Z_1_-haplotype. In summary — since all males carry the maternal Z_3_ haplotype — it is the combination of Z_1_/Z_3_ haplotypes that was absent from the population even though it should be found in approximately 50% of the male offspring according to Mendelian segregation. This suggests that the 2:1 sex ratio in the S5 population is a result of the lack of males possessing the Z_1_/Z_3_-genotype in the SR locus implying that this specific haplotype combination is lethal. Since the S3 population and the S5 population have different mothers, they posses different alleles on the maternal Z-gametolog, which are compatible (all genotypes are viable) and therefore the sex ratio in the S3-population is unbiased.

### Close linkage between the SD and the SR loci

If recombination between the SD and SR locus happened, the female determining gametolog of the SD locus would be paired with the SR allele that is absent from the male offspring population. Such females should only be viable if they inherited the compatible paternal SR allele. To assess the genetic distance between the SD and SR loci, we used all 24 female offspring that showed signs of a single maternal recombination event within the SD/SR haplotypes. We then grouped them by all possible recombination points or recombination regions and analyzed if any of the paternal haplotypes were significantly over-represented in these groups (Fishers exact test, *p* < 0.05). Since none of these tests were significant, our data did not provide evidence for the presence of female offspring with maternal recombination between the SD and SR locus. Similarly, as no male offspring with the full paternal Z_1_ haplotype exists, there is no evidence for maternal recombination between the SD and SR in male offspring. We can thus state that we did not observe recombination between the SD and the SR loci in the S5 population with 271 offspring, indicating that the linkage between the loci is tight. The linkage might even be complete, suggesting that the gametologous region not only contains the SD locus but also the SR locus. Conceivably, a specific (compatible) SR allele could be located on the W-gametolog while multiple Z-gametologs with either compatible or incompatible SR alleles exist.

### An overdominance locus could maintain the sex ratio distorter allele

**Fig. 3:**
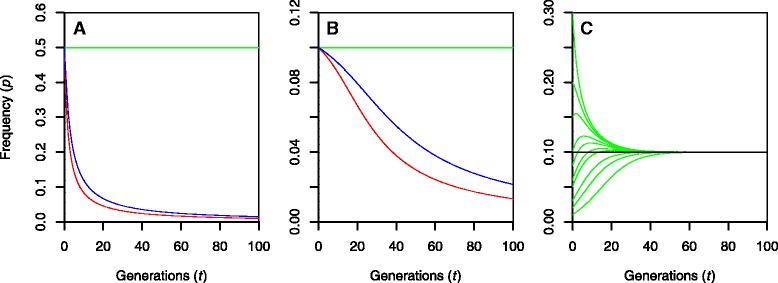
Simulated development of the frequencies of lethal/incompatible alleles/haplotypes during 100 generations (*t*) of random mating given an overdominance model (*green line*), a sex-neutral pseudooverdominance model (*red line*) or a sex-dependent pseudooverdominance model (*blue*). Scenarios included simulations of two alleles (**a**) and ten alleles (**b**) where all alleles, whether lethal/incompatible or not, exhibited equal initial allele frequencies (0.5 and 0.1 respectively). The pseudooverdominance model for two alleles is in effect equivalent to a model with a single lethal recessive allele. The stability of the overdominance model was further assessed by performing a simulation of ten alleles where initial frequencies varied (**c**) spanning a range from 0.01 to 0.3 at *t* = 0. Note: The frequency of the W-gametolog is discounted from allele frequencies of the sex-dependent models and sex-dependent and sex-neutral versions of the overdominance model behaved identically

Given that sex ratios differed between the S5 and S3 populations and that the female biased sex ratio in population S5 was caused by the absence of a genotype class among males, we hypothesize that the biased sex ratio in willows and its variation among crosses is explained by certain alleles or haplotypes causing either pre- or postzygotic incompatibility at homozygosity. To assess the stability of such alleles through many generations of random mating, we performed simplistic simulations taking into account potential allele frequency attrition as a result of incompatibility. The possibility that the W-gametolog would be devoid of any alleles causing incompatibility was also considered (sex-dependent models) as a contrast to the situation where incompatibility factors could be attached to both W- and Z-gametologs (sex-neutral models). The simplest genetic model that could possibly explain the observations of this study would be the segregation of a lethal recessive allele being situated at the sex ratio distorter locus. Iterative simulations showed however that such an allele would have a strong negative impact on reproductive success and should thus be purged from the population rapidly despite full recessivity (Fig. 3a) and irrespective of whether a sex-neutral or sex-dependent model was used.

**Fig. 4:**
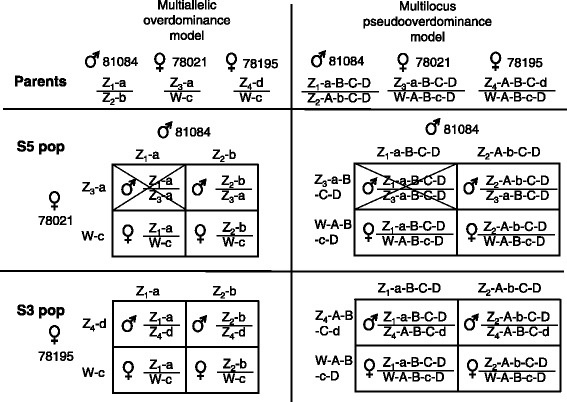
Conceptual figure showing how the multiallelic overdominance model (*left*) and multilocus pseudooverdominance model (*right*) work to make the parents (**a**) produce the marker and sex segregation patterns observed in the S5 (**b**) and S3 populations (**c**). Potentially lethal alleles are coded as small letters (e.g., Z-a-b-c-d) in contrast to alleles of with no adverse effect (Z-A-B-C-D). Sex-marker genotype combinations not observed and potentially being caused by incompatible/lethal genotypes are crossed over. Note: The particular examples in this figure show a sex-neutral version of the model where the W- as well as Z-gametologs may be associated with lethal alleles

Instead, we suggest the SR locus to be either a multiallelic overdominant effect locus in tight linkage with the SD locus (Fig. 4), or that the SR region contains several loci potentially featuring recessive lethal alleles in tight linkage with each other and the SD locus (so called *pseudooverdominance*). Both the multiallelic overdominance and multilocus pseudooverdominance models could explain the sex ratio bias and its variation among the S5 and the S3 populations and could furthermore explain the missing genotype noted for male offspring in the S5 population. Simulations illustrated that the overdominance model would perfectly preserve allele frequencies over 100 generations (Fig. 3). Stability was complete irrespective of whether two or ten alleles were simulated (Fig. 3a-b) and irrespective of whether sex-neutral or sex-dependent models were used. Stability was furthermore not affected in the long term by variable allele frequencies at the outset (Fig. 3c). The allele frequency stability is explained by the fact that only individuals heterozygous at the overdominance locus would be rendered viable. Given that the population itself survives, severe depletion or allele loss would thus become impossible. The second multilocus pseudooverdominance model was, in contrast, not strictly stable even when ten haplotypes were considered (Fig. 3b). This was mainly as a result of rare recombination events eventually producing a haplotype devoid of lethal recessive alleles. The rate of allele depletion was nonetheless slow, especially for the sex-dependent scenario, thus indicating that minor alterations (e.g., by added mutations or true overdominance) would be sufficient in order to achieve stability. Interestingly, the sex-dependent model versions exhibited overall female biased sex-ratios. Given overdominance, the frequency of females was found to be stable across generations at 67 and 53% for two alleles and ten alleles respectively. The corresponding pseudooverdominance models showed similar biases although these decreased asymptotically towards the equal sex ratio as the alleles causing incompatibility were depleted.

## Discussion

In this study, we have undertaken an in-depth segregation analysis of genome-wide SNP-alleles and compared the segregation pattern between two contrasting pedigree populations, one with even and one with female biased sex ratio. We found sex associated markers (the SD locus) on chromosome 15 in both populations, which were located in gametologous genomic regions confirming previous findings that females are the heterogametic sex in *S. viminalis* [26]. All female offspring in both populations inherited the W-gametolog from their mother while all male offspring inherited the maternal Z-gametolog (Z_3_ in the S5 population).

By contrasting the segregation pattern of paternal markers between the two populations, we discovered significantly distorted markers primarily in the S5-population with biased sex ratio. Interestingly, these markers were predominantly located in a sex ratio distorter (SR) locus close to the SD locus on chromosome 15. By analyzing the transmission of haplotypes, we were able to identify the cause of the female biased sex ratio in population S5. We discovered that all male offspring had the paternal Z_2_-haplotype in the SR locus. In contrast, the other paternal Z_1_-haplotype was never encountered although it was, as expected from Mendelian segregation, found in 50% of the female offspring. As a consequence, a 2:1 female biased sex ratio arises due to the absence of 50% of expected males with the Z_1_/Z_3_-genotype in the SR locus. No missing male genotype was found in the population with even sex ratio.

The markers in the SD locus were maternally segregating, whereas the distorted markers in the SR locus were paternally segregating. As a consequence, the relative position of these two marker types in the genetic map was dependent on nearby bi-parentally segregating markers not present within the SD/SR region. We therefore used maternal recombinant haplotypes to investigate the relative position of the SD and the SR loci. Interestingly, we did not find evidence for recombination events between the SD and the SR locus, suggesting that the two loci are tightly linked in the S5-population. This even raises the possibility that the gametologous region not only contains the SD locus but also the SR locus. A specific (compatible) SR allele is therefore likely located on the W-gametolog while multiple Z-gametologs with either compatible or incompatible alleles in the SR locus exist.

Our data thus support that the Z_1_ and the Z_3_ alleles are incompatible in the sense that this allelic combination leads to death of male offspring during their development. Alternatively the elimination may happen before fertilization if the allelic incompatibility prevents fusion of the sperm and egg. We are however unable to distinguish between these two mechanisms, since we have no exact information on when the sex bias arise. This is because sexing and genotyping was done on adult individuals, which means that we cannot strictly determine if the allelic incompatibility is pre- or postzygotic and if postzygotic, if it happens at the zygote, seed or seedling stage. The plants were however transferred to the field at recorded positions at an early age (2–3 months) and several years after planting, very few plants had died, which suggests that the Z_1_/Z_3_-males were eliminated early, possibly before or at the seed set. This is furthermore supported by previous studies on *S. viminalis* [35] and *S. repens* [31], which showed that germination approached 100% for most crosses and postgermination mortality was near 0%, suggesting that the sex bias was determined early. Similarly, in five alpine willow species it was demonstrated that the bias was not a consequence of ecological processes acting on established adult plants but rather determined at an early life stage [32]. As we have demonstrated that the biased sex ratio is the result of the absence of a male genotypic class while females are present in two classes and follow Mendelian segregation patterns, we can firmly reject the hypothesis that meiotic drive is causing the sex bias. Certation can furthermore not explain the observed genotype composition as both paternal alleles are found at similar rate in females and thus differential performance of pollen based on their genotype can be excluded.

A possible explanation to the missing male genotype is that the incompatibility allele has a recessive mutation, that in homozygous state leads to lethality of male offspring. Recessive lethal mutations are a well known phenomenon that has been shown to remove certain genotypes from a population [40–42]. However, isolated recessive lethal alleles at large frequencies would have a strong negative impact on reproductive success and should be purged to lower frequencies in very short time. Admittedly, such alleles could still have an impact if the population was subjected to inbreeding thus increasing the probability of generating homozygous individuals. But the coancestry coefficient of the S5 parents was very low in turn indicating low inbreeding in the S5 population and therefore a very low likelihood of generating genotypes homozygous for classical recessive lethal alleles. Instead, we suggest our observations to be explained by a number of incompatible alleles/haplotypes, either exhibiting overdominance or pseudooverdominance effects because such a set of alleles could be maintained in the populations over longer periods of time. Such loci, containing potentially incompatible haplotypes closely linked to the SD locus could thus explain the variation in sex ratios among offspring populations generated from different crosses [35]. Given that such loci would be associated only with the Z-gametolog (sex dependent model), such models could even explain the generally observed female biased sex ratio.

It should be noted that the concept of overdominance and pseudooverdominance effect loci regulating allelic compatibility and offspring generation is not new. Similar systems have been shown to repress the offspring generation from self crosses in several plant species (self incompatibity locus). Incompatibility can happen as late as at the moment of fertilization or even at the postzygotic stage (late-acting incompatibility [19]) raising the possibility of the abortion of incompatible haplotype combinations. Although incompatibility systems are usually designed to prevent the mating of relatives and the generation of inbred offspring, they may still occasionally be triggered in a mating of non-related parents just due to an unfortunate combination of a limited number of haplotypes/alleles. It can also be argued that dioecious species like *S. viminalis* would have no use of selfing incompatibility [43]. Nonetheless, *S. viminalis* may have retained an obsolete and partially functional self-incompatibility system, which could have been crucial in the days before dioecy was fully evolved. Indeed, a recent study on the androdioecious plant *Phillyrea angustifolia* has suggested that self-incompatibility systems can play an important role for the transition process from hermaphroditism to dioecy as well as contributing to biased sex-ratios [18].

We also considered a system of haplotypes containing several loci with lethal recessive alleles in close repulsion linkage (pseudooverdominance) since such effects have been previously observed in other studies of allele mediated lethality, usually connected with inbreeding depression [44]. However, as explained previously, inbreeding in the S5 population appeared unlikely and, with respect to the pseudooverdominance model, our simulations showed that long-term stability of such a system would require an additional factor to preserve the frequencies of the incompatibility alleles over longer time periods. It is not yet clear what this factor could be. In general the nature of the forces leading to overdominance or pseudooverdominance selection in this species system remains to be understood. The role of incompatibility mechanisms in establishing biased sex ratios is hitherto poorly studied.

## Conclusions

In conclusion, we showed that the 2:1 female biased sex ratio in a *S. viminalis* pedigree population is caused by the absence of a specific allelic combination in male offspring. This can be attributed to a lethal interaction between maternal and paternal alleles which leads to incompatibility. We demonstrate that such a system for sex ratio distortion can be stable over evolutionary times. Additionally, we also confirm previous findings of single locus sex determination and female heterogamety in *S. viminalis* even in a population with strong female biased sex ratio.

## Methods

### Plant material

The plant material used in this study originates from controlled crosses between the *S. viminalis* accessions 81084 (father in S3 and S5), 78021 (mother in S5) and 78195 (mother in S3). The pedigree population called S5 was generated in 2004 and planted the same year in a fenced area with sandy soil close to Uppsala, Sweden (59.805° N, 17.672° E) and cut down regularly at 2–4 year intervals. Sex was recorded twice in consecutive years (May 2013 and April 2014). The S3 pedigree population was first described in Höglund et al. 2005 [37] and has also been used and extended in additional studies [26, 36, 38]. In this study, we used genotype information from 516 individuals at 271 marker positions previously described in Pucholt et al. 2015 [26].

### DNA extraction

Young leaves (approximately 200 mg) from field-grown individuals were collected and snap frozen in liquid nitrogen. Samples were grinded using a TissueLyser II mill (Retsch GmbH, Haan, Germany) (4 mm steal ball, 1 min at 30 hertz). DNA was extracted following a protocol modified from Brunner et al. [45]. In brief: to every sample 950 μl of extraction buffer (100 mM TrisHCl pH 7.5–8, 25 mM EDTA, 2 M NaCl, 2% (w/v) CTAB, 2% (w/v) PVP K30, 5% (w/v) PVPP, 50 μg/ml RNAse) was added and the sample was thoroughly mixed before incubating it for 30 min at 65 °C. Subsequently 300 μl Chloroform:isoamylalcohol 24:1 was added, the sample mixed and centrifuged for 10 min at 13,000 rpm, the supernatant was transferred to a new tube and the process repeated. 1.5 volumes of icecold isopropanol was added to the supernatant followed by an incubation over night at −20 °C. After centrifugation for 10 min at 13,000 rpm at 4 °C the supernatant was removed and the pellet rinsed with cold 100% EtOH followed by another centrifugation of 5 min at 13,000 rpm at 4 °C. After that the supernatant was removed and the sample air dried before it was resolved in 100 μl TE buffer (10 mM TrisHCl, 1 mM EDTA).

### Genotyping-by-sequencing and SNP calling

Three 96-plex genotyping-by-sequencing (GBS) libraries were constructed at the Cornell University Biotechnology Resource Center (BRC) using the restriction enzyme *ApeKI* and a protocol modified from Elshire et al. [46]. Sequencing was done at the BRC Genomics Facility on Illumina HiSeq 2000/2500 instruments (100 bp, single-end reads). To call genotypes the Stacks package version 1.24 was used [47]. After demultiplexing, the reads were trimmed to 64 bp to obtain a uniform length distribution independent of the barcode size. Reads with an uncalled base or low quality scores (average below 10 in the sliding window of 15% of the read length) were removed. Genotypes were called using the denovo_map.pl module with the options -m 5 -P 5 -M 2 -n 2 -t -A CP. The rxstacks module was then used to make corrections to genotype and haplotype calls in individual samples based on data accumulated from a population-wide examination. The parameters used were --conf_filter --conf_lim 0.75 --prune_haplo --model_type snp--alpha 0.05. Subsequently the genotypes were exported using the genotypes module as onemap input file. In this step the -c option was used to automatically mitigate low confidence calls.

The data contained 579,075,921 sequencing reads from 273 individuals (271 offspring, 2 parents) with 2,039,679 ± 544,362 reads per offspring. The parents were replicated in multiple libraries to increase the reliability of genotype calls (81084: 7 libraries, 13,346,595 reads; 78021: 6 libraries, 12,976,307 reads).

Variant detection in the parental sequence yielded 41,924 potential markers that were called in the offspring. Many of these markers were not called in the majority of the offspring individuals and thus the marker set was filtered based on different criteria for the usage in the downstream analysis.

Genomic positions of all tag sequences including the markers were obtained by BLAST searches (blastn, e-value cut-off 1*10^−10^) against the *P. trichocarpa* v3 genome [48]. The best BLAST hit was assumed to represent the orthologous position of each marker in *P. trichocarpa*. The orthologous positions of the markers were located in the *Salix purpurea* genome assembly (*Salix purpurea* v1.0, DOE-JGI, http://phytozome.jgi.doe.gov/pz/portal.html#!info?alias=Org_Spurpurea) in a similar manner.

### Analyses of pattern of segregation distortion

For the S5 population, the genotypes called by Stacks were filtered for 70% per-site data completeness and separated in two groups based on reported parental genotypes. One group contained markers that were heterozygous in the father and homozygous in the mother (paternally segregating markers). The other group contained markers that were heterozygous in the mother and homozygous in the father (maternally segregating markers). In total, 3,314 paternally and 3,118 maternally segregating markers were found. Genomic positions were obtained for 2,942 paternally and 2,765 maternally markers by BLAST searches of the tag sequences against the *P. trichocarpa* genome. All markers in both groups were tested with Fisher’s exact test if the distribution of genotypes in the offspring was significantly different between sexes (Null hypothesis: genotypes are found with the same relative frequency in both sexes). The *p*-values were corrected for multiple testing within the group using Bonferroni.

Additionally the data from the S3 population [26, 36, 37] was analyzed in the same way. The dataset comprised genotypes from 137 markers located on all 19 chromosomes from 516 individuals.

### Linkage map creation and comparative mapping

The genotypes called by Stacks were filtered in two different ways for linkage map creation. In the basic dataset, loci were retained that were called in at least 250 individuals (92%), with a maximum genotype frequency below 60% and a *p*-value larger than 0.0005 for segregation distortion in the whole population. With these filter criteria we obtained 4217 high confidence sites. In an extended dataset, loci were retained that were called in at least 90 individuals (33%), with a maximum genotype frequency below 70% and a *p*-value larger than 0.0005 for segregation distortion in the whole population. This extended dataset contained 10,546 sites. The filtration on segregation distortion was applied since sequencing errors that are present at low frequency behave similar to alleles with extreme segregation distortion. Additionally to the genetic markers, sex was added as a morphological marker. Both a paternal and a maternal segregation of this morphological marker was tested, but it could only be positioned in the linkage map when maternal segregation was assumed.

Linkage maps were calculated based on the basic genotype dataset using the R package “onemap” version 2.0-4 [49] applying a threshold of logarithm of odds (LOD) = 7 and maximum recombination fraction of 0.3 for marker grouping and the Kosambi mapping function to estimate genetic distances. Marker order was calculated using the order.seq function within onemap with an initial set of six markers (seven markers for chromosome 8) and the “touchdown” option activated. Only markers that could be assigned a unique position on the map were used. Linkage groups were assigned chromosome IDs by BLAST searches of the tag sequences of the markers within that group to the *P. trichocarpa* genome and selecting the ID that the majority of markers agreed on (Additional file 2).

The mapping position of the morphological marker for sex defined the sex determination locus. To increase the density of markers in proximity to the “sex” marker, additional markers from the extended dataset were integrated into this map. The two genetic markers positioned adjacent to the “sex” marker in the basic map, “133572” and “27372”, were used as landmarks to identify additional markers. These additional markers were selected as either mapping (blastn best hit, evalue < 1*10^−10^) to at least one of the genomes of *P. trichocarpa* [48] or *S. purpurea* (*Salix purpurea* v1.0, DOE-JGI, http://phytozome.jgi.doe.gov/pz/portal.html#!info?alias=Org_Spurpurea) between the landmark markers or as mapping to the Scaffold 64 in *S. suchowensis* [50]. This set of markers was separated into linkage groups with the parameters stated above to exclude markers for which the location within the linkage group of the incipient sex chromosome was not supported by our data. One main linkage group was found and several unlinked markers and small separated linkage groups were excluded. A new map of the sex chromosome was constructed and used as representative of chromosome 15 in the further analysis. The final genetic map of 1,976 markers had a total size of 2,808.29 cM in 19 linkage groups and covered 94.8% of the *P. trichocarpa* genome.

Macrosyntheny between the *S. viminalis* genome and the *P. trichocarpa* genome was assessed by comparing the position of markers in the map and the predicted orthologous position (best BLAST hit) of these markers in the *P. trichocarpa* genome. The previously described chromosomal rearrangement involving chromosome 1 and 16 [36] was confirmed and we showed that *S. viminalis* chromosome 16 consists of *P. tricocarpa* chromosome 16 and a part of chromosome 1 (Additional file 2) and the remaining part of chromosome 1 of *P. tricocarpa* is represented by a different linkage group in *S. viminalis*. Apart from these, extensive macrosynteny was demonstrated which allows us to apply *P. tricocarpa* based positions in our analyses (Additional file 2).

### Haplotype inference and segregation analysis

Based on the observation of segregation distortion of marker alleles on chromosome 15, we performed a more detailed analysis of the region in the linkage map spanning 54.5 to 75.8 cM on chromosome 15. The markers in this region were supplemented with markers that were significantly distorted (and therefore not included in the linkage map) and that had best BLAST hits in the *S. purpurea* genome between the map markers flanking the region of interest on chromosome 15. The order of the markers in the *S. purpurea* draft genome was used and the approximate positions of the markers were calculated by positioning the additional markers equidistant on the map. In this way, for the maternal haplotypes, seven markers in the consensus genetic map of chromosome 15 were supplemented with 33 additional markers and for the paternal haplotypes, three markers in the consensus genetic map of chromosome 15 were supplemented with thirteen additional markers.

The genotypes at the markers were phased to predict haplotypes by minimizing the number of recombination events between adjacent markers based on genotype data from the full population. This phase information was then used to determine which individuals that carried recombinant or pure parental haplotypes and which of the parental haplotypes they had inherited.

Female offspring with maternal recombination events were identified as those female individuals that had one transition between the maternal haplotypes in the studied region. For statistical analysis of over-representation of certain genotypes, all female offspring with a maternal recombination event were grouped by their recombination points. The groups consisted of all individuals that had one haplotype in the marker 1 to *n* and the other haplotype in the markers *m* to 40, with *n* and *m* ∈ {1…40}. Fishers exact test without correction for multiple testing was used to analyze if, for any of the groups, one of the paternal haplotypes was over-represented.

### Genetic models

In order to explain the segregation of the sex determination and the sex ratio distorter locus, we proposed two genetic models which should produce a reasonably stable sex ratio and allele frequencies over generations. The first *multiallelic overdominance model* features the Z/W sex determination locus at chromosome 15 being tightly linked to a multiallelic locus for which any homozygote allele combination will cause either pre- or postzygotic allelic incompatibility (Fig. 4). In contrast, the second *multilocus pseudooverdominance model* link the sex determination locus to a number of tightly linked biallelic loci exhibiting recessive lethal effects. Because each mutation is associated with a distinct haplotype, these recessive mutations will frequently produce a seemingly overdominant behaviour. Therefore, in spite of the contrasting architecture of these models, they will generally produce segregation patterns that are similar in nature.

The stability of these two models was assessed by performing simple iterative simulations (shown in detail in Supplementary Materials and Methods in Additional file 3). These simulations assumed a constant infinite population size, random mating and complete linkage between sex determination and ratio loci. The most important difference between the multiallelic overdominance and multilocus pseudooverdominance models was that the latter featured an allele without the lethal effect at homozygosity (e.g., Z-A-B-C-D, Fig. 4). In a multilocus setting it is likely that recombination sooner or later would produce a haplotype free of mutations and this possibility had to be accounted for. Both overdominance and pseudooverdominance genetic models were furthermore each subdivided into two versions: (i) a *sex-neutral* version where alleles causing lethality or incompatibility could be present in both Z- and W-gametologs and will thus behave as autosomally inherited alleles; and (ii) a *sex-dependent* version where lethal alleles are associated with the Z-gametolog only and where hemizygous females consistently are regarded as heterozygous for the sex distorter locus thus always being unaffected.

Simulation included a scenario featuring only two alleles and another scenario with ten alleles. For the two-allele scenario, both alleles could be lethal at homozygosity (overdominance model) or one of the alleles could be recessive lethal while the other dominant allele would have no adverse effect. Correspondingly, in the ten-allele scenario the overdominance model would assign lethality at homozygosity to all alleles while for the pseudooverdominance model, nine haplotypes would be potentially lethal while the tenth would always be harmless.

## Additional files

Additional file 1: Genetic markers with segregation distortion. Table of genetic markers showing segregation distortion in the S5 pedigree population with biased sex ratio. The sequence tag surrounding the marker is given as well as the position of the homologous sequence in *P. trichocarpa*, the number of individuals in the population with each genotype, separated by their gender and the corrected *p*-value. (XLS 34 kb)

Additional file 2: Genetic map and syntheny analysis. *S. viminalis* whole genome genetic map and analysis of syntheny with the *P. trichocarpa* genome sequence. (PDF 200 kb)

Additional file 3: Supplementary genetic model description. (PDF 46 kb)

## Abbreviations

BRC: Cornell University Biotechnology Resource Center
GBS: Genotyping-by-sequencing
LOD: Logarithm of odds
SD: Sex determination
SNP: Single nucleotide polymporphism
SR: Sex ratio distorter
