# Supplementary material for: Allelic incompatibility can explain female biased sex ratios in dioecious plants
Source: BMC Genomics. 2017 Mar 23;18:251. doi: 10.1186/s12864-017-3634-5 (PMC5364565; doi:10.1186/s12864-017-3634-5)
Supplement: Supplementary file 2 — Genetic map and syntheny analysis. S. viminalis whole genome genetic map and analysis of syntheny with the P. trichocarpa genome sequence. (PDF 200 kb) [file 12864_2017_3634_MOESM2_ESM.pdf]

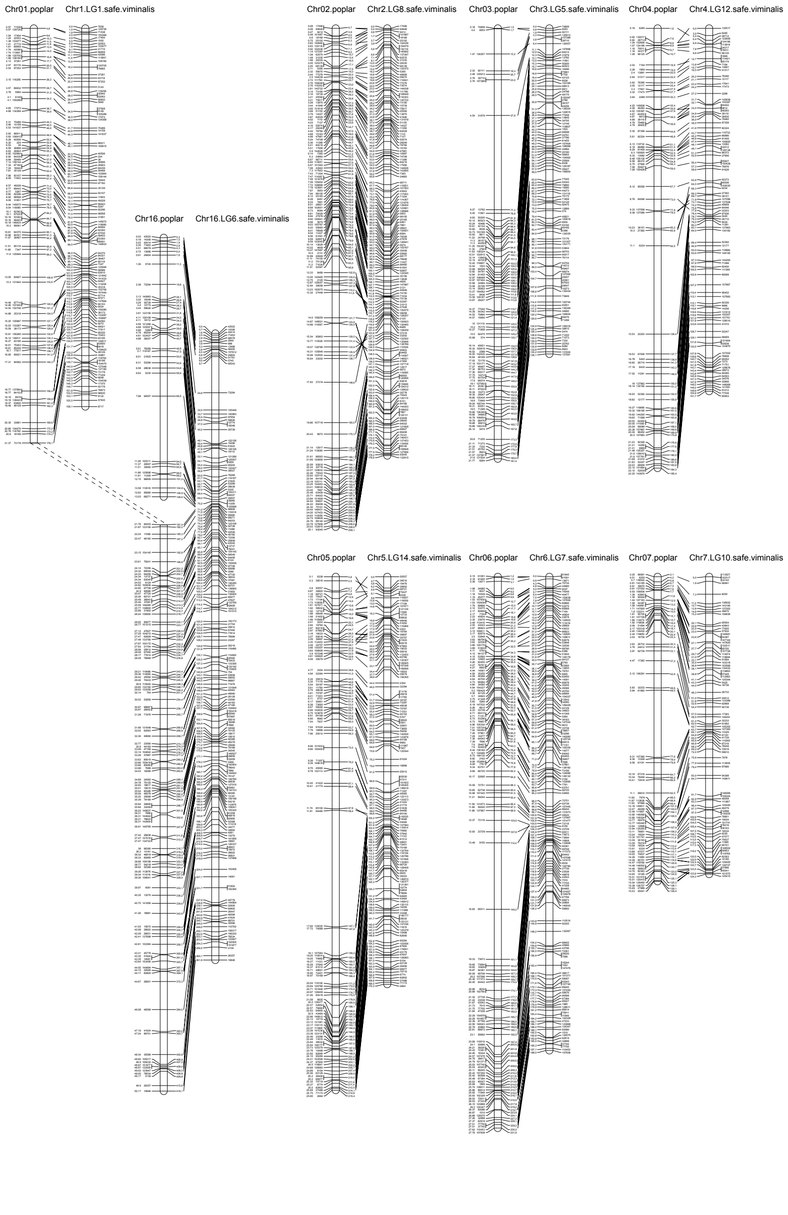

Figure 1: Linkage groups representing chromosomes 1 to 7 and 16 in *S. viminalis*. To the left of each genetic map the *P. trichocarpa* genome is shown with the positions of the markers used for the genetic map and connecting lines between the maps of the two species. Centimorgan positions for the markers in the genetic map are given to the left of the *S. viminalis* map and physical positions of the markers in Mbp are given leftmost beside the *P. trichocarpa* map.

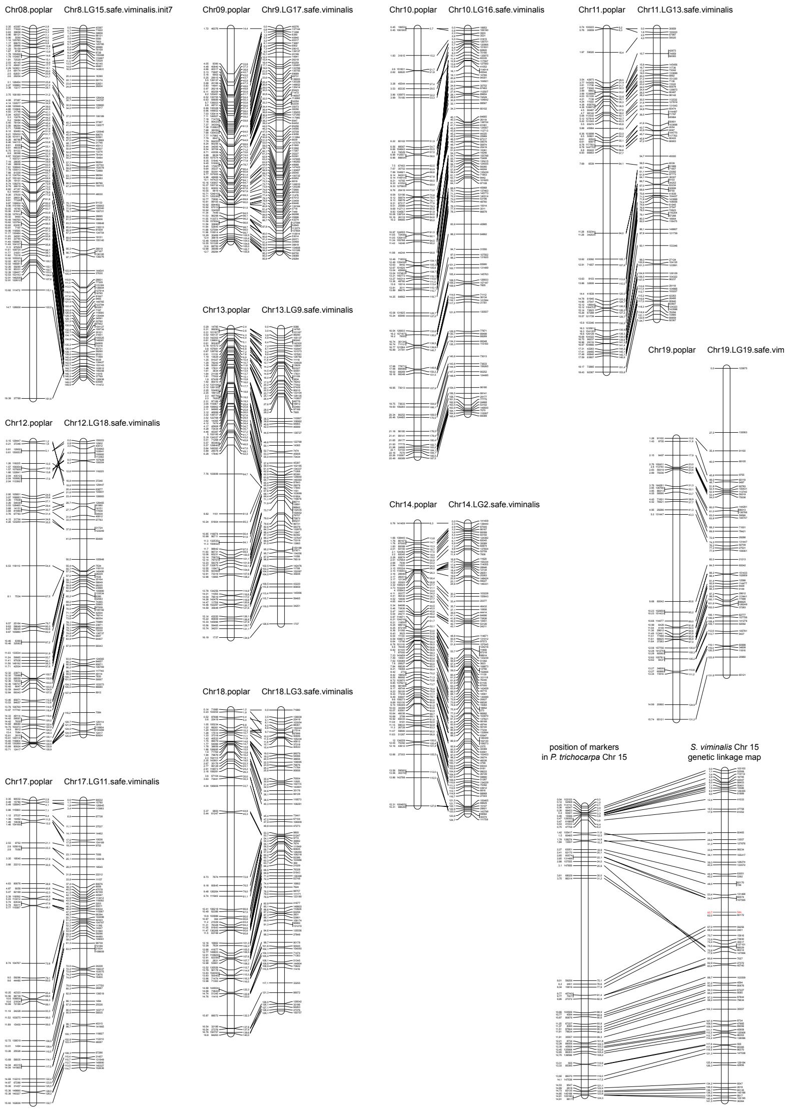

Figure 2: Linkage groups representing chromosomes 8 to 15 and 17 to 19 in *S. viminalis*. To the left of each genetic map the *P. trichocarpa* genome is shown with the positions of the markers used for the genetic map and connecting lines between the maps of the two species. Centimorgan positions for the markers in the genetic map are given to the left of the *S. viminalis* map and physical positions of the markers in Mbp are given leftmost beside the *P. trichocarpa* map.
