# Supplementary material for: Allelic incompatibility can explain female biased sex ratios in dioecious plants
Source: BMC Genomics. 2017 Mar 23;18:251. doi: 10.1186/s12864-017-3634-5 (PMC5364565; doi:10.1186/s12864-017-3634-5)
Supplement: Supplementary file 3 — Supplementary genetic model description. (PDF 46 kb) [file 12864_2017_3634_MOESM3_ESM.pdf]

Supplementary Material & Methods 3

Allelic incompatibility can explain female biased sex ratios in dioecious plants

Pascal Pucholt, Henrik R. Hallingbäck, Sofia Berlin

Genetic model description

Since the sex-dependent version of the genetic models is more complicated than the sex-neutral version we base the following descriptions on the sex-dependent version and subsequently state how the sex-neutral version can be obtained by simplification. Let us consider a population exhibiting any of the alleles N_i where $i = 1 \ldots n$ at a single locus N within the Z-gametolog (multiallelic overdominance model) or as haplotypes N_i each with a single recessive lethal allele at any of the tightly linked loci $i = 1 \ldots n$ within or close to the Z-gametolog (multilocus pseudooverdominance model). For the pseudooverdominance model we moreover introduce a haplotype that did not contain any lethal alleles (A) and in the sex-dependent version we also assumed that the W-gametolog (W) would have no lethal alleles thereby consistently counteracting eventual lethal alleles in the Z-gametolog. We disregard any haplotypes with lethal alleles at multiple positions within the haplotype as they would confer considerable fitness disadvantages and we assume that no further recombination takes place in the short term. We also assume random mating with the restriction that males can only mate females and vice versa and that sex biases will have no impact on total fertilization and seed production.

For the overdominance model we should then be able to calculate the frequencies of two possible genotypes: (i) males with two different haplotypes/alleles ($p(N_i N_j)$) lethal when homozygous and (ii) females with one such haplotype/allele being complemented by the W-gametolog ($p(N_i W)$). However for the pseudooverdominance model there will be additional genotypes to consider: (iii) males with only one lethal haplotype/allele ($p(N_i A)$); (iv) males with two haplotypes devoid of lethal alleles ($p(AA)$) plus; (v) corresponding females devoid of lethal alleles/haplotypes ($p(AW)$). All allele/haplotype frequencies will have to be accounted for separately for each sex (i.e. $p(N_{i♀})$, $p(N_{i♂})$, $p(A_♀)$, $p(A_♂)$) while $p(W)$ will always be associated with the female part of the population. At this juncture it should be remembered that the total frequency of females, and consequently also the corresponding frequency of males, has a simple relationship to the frequency of W-alleles ($p(♀) = 2p(W)$ and $p(♂) = 1 - 2p(W)$). It is then shown that the frequency of the genotype $N_i N_j$ at generation $t+1$ can be

calculated from the allele frequencies of the parental generation t as:

$$p(N_iN_j)_{t+1} = \frac{\frac{p(N_{i♀})_t p(N_{j♂})_t}{p(♀)_t p(♂)_t} + \frac{p(N_{j♀})_t p(N_{i♂})_t}{p(♀)_t p(♂)_t}}{1 - \sum_{k=1}^{n} \frac{p(N_{k♀})_t p(N_{k♂})_t}{p(♀)_t p(♂)_t}} = \frac{p(N_{i♀})_t p(N_{j♂})_t + p(N_{j♀})_t p(N_{i♂})_t}{2p(W)_t(1-2p(W)_t) - \sum_{k=1}^{n} p(N_{k♀})_t p(N_{k♂})_t} \quad (1)$$

Observe that all the probabilities has to be scaled by the frequencies of males and females present in the parental population in order to reflect eventual differences in allele frequencies between the sexes. Moreover the regular Hardy-Weinberg like allele frequency proportions have to be adjusted to discount all genotypes homozygous for the lethal haplotypes $(p(N_{k♀})_t p(N_{k♂})_t / p(♀)_t p(♂)_t)$ because those genotypes are to be removed from the pool of surviving genotypes. In summary, the frequencies of all possible genotypes in generation t+1 are calculated as the first step of a generation iteration and can be simplified as follows:

Step1 :

$$\begin{aligned}
p(N_iN_j)_{t+1} &= \frac{p(N_{i♀})_t p(N_{j♂})_t + p(N_{j♀})_t p(N_{i♂})_t}{2p(W)_t(1-2p(W)_t) - \sum_{k=1}^{n} p(N_{k♀})_t p(N_{k♂})_t} \\
p(N_iW)_{t+1} &= \frac{p(W)_t p(N_{i♂})}{2p(W)_t(1-2p(W)_t) - \sum_{k=1}^{n} p(N_{k♀})_t p(N_{k♂})_t} \\
p(N_iA)_{t+1} &= \frac{p(N_{i♀})_t p(A_{♂})_t + p(A_♀)_t p(N_{i♂})_t}{2p(W)_t(1-2p(W)_t) - \sum_{k=1}^{n} p(N_{k♀})_t p(N_{k♂})_t} \\
p(AA)_{t+1} &= \frac{p(A_♀)_t p(A_♂)_t}{2p(W)_t(1-2p(W)_t) - \sum_{k=1}^{n} p(N_{k♀})_t p(N_{k♂})_t} \\
p(AW)_{t+1} &= \frac{p(W)_t p(A_♂)_t}{2p(W)_t(1-2p(W)_t) - \sum_{k=1}^{n} p(N_{k♀})_t p(N_{k♂})_t} \quad (2)
\end{aligned}$$

with the restrictions that $i \neq j$ and $p(N_iN_i)_{t+1} = 0$ for all i and t. Subsequently we should consider that the new allele frequencies at generation t+1 are:

Step2 :

$$\begin{aligned}
p(N_{i♂})_{t+1} &= \frac{1}{2}(p(AN_i)_{t+1} + \sum_{j=1}^{n} p(N_iN_j)_{t+1}) \\
p(N_{i♀})_{t+1} &= \frac{1}{2} p(N_iW)_{t+1} \\
p(A_♂)_{t+1} &= p(AA)_{t+1} + \frac{1}{2}\sum_{j=1}^{n} p(N_jA)_{t+1}
\end{aligned}$$

$$
\begin{aligned}
p(A_{\female})_{t+1} &= \frac{1}{2}p(AW)_{t+1} \\
p(W)_{t+1} &= \frac{1}{2}(p(AW)_{t+1} + \sum_{j=1}^{n} p(N_j W)_{t+1})
\end{aligned} \tag{3}
$$

It is then possible to advance the simulation by iteratively repeat steps 1 and 2 until a desirable number of generations have passed. In their entirety, these formulae describe the chief properties of the multilocus pseudooverdominance model. The multiallelic overdominance model can simply be obtained by assuming the absence of a mutation-free allele (i.e. $p(A_{\male})_t = p(A_{\female})_t = 0$ for all t). Moreover, in a sex-neutral model,the W-gametolog is no longer considered to function as a allele itself and can thus also contain lethal alleles/haplotypes. In such a system, sex can be completely ignored as a factor thus simplifying step 1 calculations to:

$$
\begin{aligned}
p(N_i N_j)_{t+1} &= \frac{2p(N_i)_t p(N_j)_t}{1 - \sum_{k=1}^{n} p(N_k)_t^2} \\
p(N_i A)_{t+1} &= \frac{2p(N_i)_t p(A)_t}{1 - \sum_{k=1}^{n} p(N_k)_t^2} \\
p(AA)_{t+1} &= \frac{p(A)_t^2}{1 - \sum_{k=1}^{n} p(N_k)_t^2}
\end{aligned} \tag{4}
$$

Step 2 calculations for $p(N_i)_{t+1}$ and $p(A)_{t+1}$ will follow that of $p(N_{i\male})_{t+1}$ and $p(A_{\male})_{t+1}$ respectively in eq. 3.
